# Myeloid-Derived Suppressor Cells in *Trypanosoma cruzi* Infection

**DOI:** 10.3389/fcimb.2021.737364

**Published:** 2021-08-27

**Authors:** Manuel Fresno, Núria Gironès

**Affiliations:** ^1^Centro de Biología Molecular Severo Ochoa, Consejo Superior de Investigaciones Científicas, Universidad Autónoma de Madrid, Cantoblanco, Madrid, Spain; ^2^Instituto de Investigación Sanitaria del Hospital Universitario de La Princesa, Group 12, Madrid, Spain

**Keywords:** myeloid-derived suppressor cells, *Trypanosoma cruzi*, arginase 1, inducible nitric oxide synthase, L-arginine, SLAMF1, ROS, RNS

## Abstract

Myeloid-derived suppressor cells (MDSCs) are immature heterogeneous myeloid cells that expand in pathologic conditions as cancer, trauma, and infection. Although characterization of MDSCs is continuously revisited, the best feature is their suppressor activity. There are many markers for MDSC identification, it is distinctive that they express inducible nitric oxide synthase (iNOS) and arginase 1, which can mediate immune suppression. MDSCs can have a medullary origin as a result of emergency myelopoiesis, but also can have an extramedullary origin. Early studies on *Trypanosoma cruzi* infection showed severe immunosuppression, and several mechanisms involving parasite antigens and host cell mediators were described as inhibition of IL-2 and IL-2R. Another mechanism of immunosuppression involving tumor necrosis factor/interferon γ-dependent nitric oxide production by inducible nitric oxide synthase was also described. Moreover, other studies showed that nitric oxide was produced by CD11b^+^ Gr-1^+^ MDSCs in the spleen, and later iNOS and arginase 1 expressed in CD11b^+^Ly6C^+^Ly6G^lo^ monocytic MDSC were found in spleen and heart of *T. cruzi* infected mice that suppressed T cell proliferation. Uncontrolled expansion of monocytic MDSCs leads to L-arginine depletion which hinders nitric oxide production leading to death. Supplement of L-arginine partially reverts L-arginine depletion and survival, suggesting that L-arginine could be administered along with anti-parasitical drugs. On the other hand, pharmacological inhibition of MDSCs leads to death in mice, suggesting that some expansion of MDSCs is needed for an efficient immune response. The role of signaling molecules mediating immune suppression as reactive oxygen species, reactive nitrogen species, as well as prostaglandin E2, characteristics of MDSCs, in *T. cruzi* infection is not fully understood. We review and discuss the role of these reactive species mediators produced by MDSCs. Finally, we discuss the latest results that link the SLAMF1 immune receptor with reactive oxygen species. Interaction of the parasite with the SLAMF1 modulates parasite virulence through myeloid cell infectivity and reactive oxygen species production. We discuss the possible strategies for targeting MDSCs and SLAMF1 receptor in acute *Trypanosoma cruzi* infection in mice, to evaluate a possible translational application in human acute infections.

## MDSCs

The existence of populations of immature myeloid suppressor cells was described in different murine models of immune suppression and cancer (Angulo et al., 2000; Kusmartsev et al., 2000), among others, and associated with the production of nitric oxide (NO) and other reactive nitrogen species (RNS). To distinguish them from normal immature myeloid cells (IMCs) that give rise to granulocytes, macrophages, and dendritic cells, they were named as myeloid-derived suppressor cells, and abbreviated first as MSCs (Sinha et al., 2005) and later as MDSCs (Gabrilovich et al., 2007). MDSCs are a heterogeneous population of CD11b^+^Gr-1^+^ cells including myeloid progenitors and IMCs that expand and accumulate during tumor growth, trauma, acute and chronic immune responses to pathogens, and other altered immune responses (Gabrilovich and Nagaraj, 2009). However, this population is best defined by their suppressor activity (Bronte et al., 2016), which differentiates them from normal IMCs. MDSCs show important phenotypic differences depending on the anatomical site where they are located or the pathological condition. In addition, in tumor-induced MDSCs, discrete subpopulations with distinct T cell suppressive activity have been identified (Marigo et al., 2008; Movahedi et al., 2008).

### Identification of MDSCs Subsets

Initially, MDSCs were identified using anti-Gr-1 antibodies that recognized both, Ly6G and Ly6C, surface molecules. Later, the use of antibodies specific for Ly6C and Ly6G surface antigens allowed the classification of MDSCs in monocytic CD11b^+^Ly6C^+^Ly6G^lo^ (M-MDSCs) and polymorphonuclear CD11b^+^Ly6C^-^Ly6G^hi^ (PMN-MDSCs), also named granulocytic MDSCs (G-MDSCs). M-MDSCs share some features of classical (CAM or M1) and alternatively activated (AAM or M2) macrophages. M1 are induced by proinflammatory Th1 cytokines as IFN-γ, which express inducible nitric oxide synthase (iNOS). Some subsets of M2 macrophages that are induced by anti-inflammatory cytokines such as IL-4 and IL-13, express arginase 1 (Arg1). However, MDSCs express both enzymes (Gabrilovich and Nagaraj, 2009). There are other characteristics of M-MDSCs besides iNOS and Arg1, as production of prostaglandin E2 (PGE_2_), and expression of Transforming growth factor β (TGF-β), Interleukin 4 receptor α (IL-4Rα), and IL-13. On the other hand, PMN-MDSCs express RAR-related orphan receptor gamma 1 (RORC1), NADPH oxidase (NOX)-2, and granulocyte-colony stimulating factor (G-CSF). Finally, there are some markers common to both subsets as Phospho-Signal transducer and activator of transcription 3 (STAT3), CCAAT enhancer-binding protein beta (c/EBP/β), S100 calcium-binding proteins (S100A8 and S100A9), Programmed death-ligand 1 (PD-L1), IL-10, granulocyte-macrophages colony-stimulating factor (GM-CSF), IL-1, (Bronte et al., 2016), and indoleamine 2,3-dioxygenase (IDO) (Zhao et al., 2016). Because some of those markers are not exclusive of MDSCs, the best probe of *bona fide* MDSCs is their ability to suppress T cell proliferation. However, it was proposed that subsets expressing MDSC markers that do not suppress proliferation should be described as MDSC-like cells (MDSC-LC) (Bronte et al., 2016).

### Suppressor Mechanisms of MDSCs

**Figure 1:**
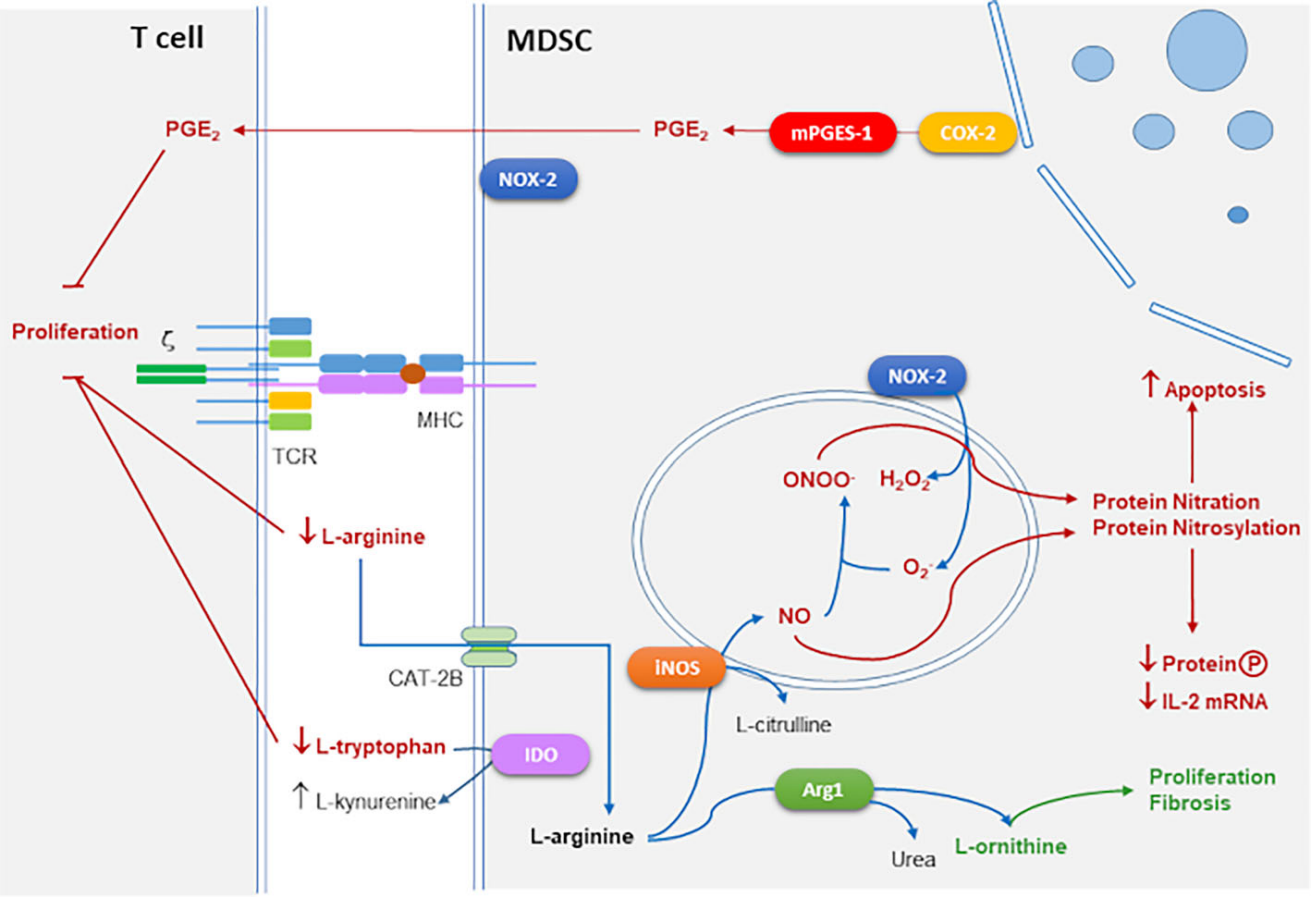
Main mechanisms of T cell immune suppression mediated by MDSCs. Suppressive action of iNOS and NADPH oxidase 2 (NOX-2) by RNS and reactive oxygen species (ROS) generation mediated by protein nitration and nitrosylation leads to apoptosis and inhibition of protein phosphorylation and IL-2 mRNA degradation. Arg1 together with iNOS consume extracellular L-arginine which inhibits CD3 ζ-chain expression interfering with TCR signaling. Arg1 produces L-ornithine that is the substrate of polyamines linked to proliferation and collagen involved in tissue repair and fibrosis. Cyclooxygenase 2 (COX-2) and microsomal prostaglandin E2 synthase (mPGES-1) produce suppressive prostaglandin E2 (PGE_2_). Indoleamine 2,3-Dioxygenase (IDO) converts L-tryptophan into L-kynurenine which also blocks T cell proliferation.

Some of the main mechanisms of suppression of T cell proliferation by MDSC are outlined in **Figure 1**.

iNOS and Arg1 use L-arginine (L-arg) as a substrate for their enzymatic activities. Extracellular L-arg is transported to the cytoplasm through cationic amino acid transporter 2B (CAT2B). iNOS converts L-arg into NO which is toxic for microbes, and citrulline. Arg1 converts L-arg into urea and L-ornithine, which in turn is converted into polyamines involved in cell growth and differentiation, and L-proline that affects collagen production, tissue repair, and fibrosis (Bronte and Zanovello, 2005). L-arg is a semi-essential amino acid in adult mammals and it is required in the diet to maintain adequate levels in some stressed conditions as pregnancy, trauma, or infection (Bronte and Zanovello, 2005). In addition, T cell proliferation is extremely sensitive to extracellular L-arg concentration. Thus, the threshold of L-arg concentration in mammalian plasma that permits fully functional T cell proliferation is in the order of 100 μM (Choi et al., 2009). Moreover, the combined activity of Arg1 and iNOS enzymes is important in the suppressive activity of mouse MDSCs in tumors (Bronte et al., 2003).

MDSCs use several mechanisms to suppress the T cell response, such as triggering apoptosis of activated T cells by depleting of L-arg, *via* Arg1 (Apolloni et al., 2000). L-arg depletion also causes translational blockade of the ζ-chain of CD3, possibly through pathways that involve the kinases general control non-depressible 2 (GCN2) and mammalian target of rapamycin (mTOR) (Bronte and Zanovello, 2005). There is evidence that T cell suppression mediated by MDSCs is antigen-specific, this is because MDSCs can take up microbial antigens, process them, and present them to CD8^+^ T cells (Movahedi et al., 2008; Youn et al., 2008), thus, MDSCs can directly suppress CD8^+^ T cells after peptide presentation by MHCI to the T cell receptor (TCR). There is also evidence that MDSCs may suppress immune activation by inducing T regulatory cell expansion (Huang et al., 2006). Other suppressive mechanisms that have been proposed include the production of TGF-β, depletion of cysteine, induction of Cyclooxygenase-2 (COX-2) expression, and PGE_2_ production (Ostrand-Rosenberg, 2010).

Besides, iNOS produces NO, which causes nitrosylation of cysteine residues in target proteins and production of cyclic GMP (cGMP) by the soluble guanylate cyclase (sGC) pathway. These events interfere with the interleukin-2 receptor (IL-2R)-signaling pathway by blocking the phosphorylation of signal-transducing molecules that are coupled to IL-2R and by altering the stability of mRNA encoding IL-2 (Bronte and Zanovello, 2005).

When L-arginine concentrations are limiting due to Arg1 activity, and iNOS levels are high, electrons can be transferred by iNOS subunits to the co-substrate oxygen (O_2_), which generates superoxide anion $\left({\text{O}}_{2}^{-}\right)$, a reactive oxygen species (ROS). Other sources of ${\text{O}}_{2}^{-}$ may come from NADPH oxidase-2 (NOX-2) activity in other MDSC subsets. NOX-2 is a multi-subunit enzyme with membrane and cytosolic components, which actively communicate during the host responses to a wide variety of stimuli and mediates ROS production, which has an important role in the elimination of invading microorganisms in macrophages and neutrophils (Panday et al., 2015) and also expressed by PMN-MDSCs (Bronte et al., 2016). ${\text{O}}_{2}^{-}$ can combine either with NO to generate peroxynitrites (ONOO^–^) or with water (H_2_O) to produce hydrogen peroxide (H_2_O_2_) (Bronte and Zanovello, 2005). Then, ONOO^–^ causes nitration of proteins in tyrosine residues interfering with multiple signals, which together with H_2_O_2_ induce apoptosis (Bronte and Zanovello, 2005).

Activation of the enzyme indoleamine 2,3-Dioxygenase (IDO), which converts L-tryptophan to L-kynurenine causes depletion of extracellular L-tryptophan which can also cause suppression of T cell proliferation (Kumar et al., 2016).

Most studies on macrophages and MDSCs are performed in mouse models, and their role in humans is frequently questioned. In particular, Arg1 is abundantly expressed in mouse M2 macrophages and MDSCs (Bronte et al., 2003), but there is controversy about their expression in humans, which seems to be mostly expressed in granulocytes (Munder et al., 2005). However, transcriptomic and phenotypic profiling studies showed similarities between human and mouse myeloid populations (Guilliams et al., 2014). Moreover, arginase activity was detected in human tuberculosis granulomas in human macrophage subsets (Mattila et al., 2013), thus it might be expressed in other cell types in pathological conditions. It is important to note that dissimilar strains of mice behave differently, macrophages from a prototypical Th1 strain such as C57BL/6, produce more NO than macrophages from a prototypical Th2 strain for example BALB/c activated with IFN-γ and LPS (Mills et al., 2000). Similarly, human individuals can respond differently to infection (Thomas and Mattila, 2014).

### Origin of MDSC

In severe systemic infections, there are profound alterations in the hematopoietic function of the BM that likely reflect a homeostatic response to a decrease in circulating immune populations (Glatman Zaretsky et al., 2014). Hematopoietic progenitors in the BM can sense the presence of pathogens through pattern recognition receptors, as Toll-like receptors (TLRs), or in response to proinflammatory cytokine stimuli leading to an increased generation of granulocyte-macrophage precursors (GMPs) and common myeloid precursors (CMPs), which then expand and further differentiate towards neutrophils facilitating what is called “emergency myelopoiesis”, at the expense of erythroid and lymphoid differentiation (Mitroulis et al., 2018), or giving rise to immature neutrophils that migrate to peripheral tissues (Evrard et al., 2018).

Although there is a consensus that all MDSC derive from the same precursors, there is a controversy about their medullary or extramedullary origin. The origin (and appearance) of MDSCs in each particular physiopathological context is different and not completely understood. Elevated levels of colony-stimulating factors (CSFs) may induce emergency myelopoiesis (Panopoulos and Watowich, 2008) that produces migration of IMCs from BM before their full differentiation, to replace cells consumed during inflammation, in particular, due to infection (Cheers et al., 1988; Kawakami et al., 1990).

The classical hypothesis to explain MDSCs generation by emergency myelopoiesis is known as the “two signal model” (Condamine et al., 2015), where signal 1 is given by GM-CSF, G-CSF, and IL-6, that mobilizes IMCs from bone marrow which is also called “emergency granulo-monocytopoiesis” (Strauss et al., 2015). Signal 2 is mainly mediated by Toll-like receptor signals that signal through NFκB for cytokine production (Gabrilovich and Nagaraj, 2009; Condamine et al., 2015). After leaving the bone marrow, IMCs can become MDSCs in the spleen, where extramedullary myelopoiesis can occur (Song et al., 2005). Thus, it is also feasible that expansion of MDSCs occurs in secondary lymphoid organs.

In the context of cancer therapy, there are many approaches to target MDSCs (reviewed by Gabrilovich, 2017) Which could be assayed for *T. cruzi* and other infectious diseases where they play a detrimental role. MDSCs can be eliminated with gemcitabine and 5-fluorouracil (5FU) chemotherapy, and inhibiting the tumor necrosis factor-related apoptosis-inducing ligand (TRAIL) receptor and S100A9. But also targeting the suppressive machinery by inhibiting phosphodiesterase type 5 (PDE-5), COX-2, and Class I Histone deacetylase (HDAC). Besides, upregulation of the nuclear factor erythroid 2-related factor 2 (Nrf2) with synthetic triterpenoids reduces MDSCs suppressive activity. Other approaches targeted MDSCs expansion and differentiation as treatment with all-trans-retinoic acid (ATRA), inhibition of STAT3, and treatment with phospholipid phosphatidylserine (PS) antibodies. In addition, microRNA networks that regulate the differentiation, expansion, and suppression function of MDSCs may provide a novel potential approach for targeting MDSCs in the tumor environment (Su et al., 2019).

## *Trypanosoma cruzi* Infection

*Trypanosoma cruzi* is a unicellular protozoan parasite that causes the Chagas disease or American Trypanosomiasis, a chronic debilitating condition endemic of Latin America, and a neglected tropical disease (Pérez-Molina and Molina, 2018). Nowadays, it has been estimated that there are around 6–7 million chronically infected people [World Health Organization Chagas Disease (American trypanosomiasis)]. This parasite has a very complex life cycle that includes an invertebrate hematophagous *Reduvidae* insect vector and multiple mammalian hosts (Clayton, 2010). Also, *T. cruzi* has different vital stages (de Souza et al., 2010; Rodrigues et al., 2014). The non-infective epimastigotes are present in the midgut of triatomines insect vectors where they differentiate into infective metacyclic trypomastigotes, that after the infection of host cells are differentiated into the intracellular replicative amastigote form. After replication amastigotes differentiate to infective trypomastigotes that reach the bloodstream after cell lysis (Echeverria and Morillo, 2019). Chagas disease presents an acute phase with low mortality and symptomatology. Then, the patients can remain in an asymptomatic chronic phase for life or in the 30–40% of cases and after 10–30 years after infection may develop chronic myocarditis and cardiomegaly, or megavisceras (megaesophagus or megacolon) or both (Rassi et al., 2012).

### Immune Suppression in *Trypanosoma cruzi* Infection

Early reports from the 70-80s showed that infection with *T. cruzi* is associated with severe unresponsiveness to mitogens and antigens during the acute phase of the disease. It was thought that a suppressor factor was the cause (Cunningham et al., 1978; Kierszenbaum, 1982). Moreover, other reports pointed out T cells (Tarleton, 1988a; Guimarães-Pinto et al., 2018), including γδ T cells (Cardillo et al., 1998), as well as adherent cells (Kierszenbaum, 1982; Motrán et al., 1996) as suppressor cells. In addition, inhibition of Interleukin (IL)-2 synthesis (Harel-Bellan et al., 1985; Tarleton, 1988a) and reduced cell surface expression of IL-2 receptor by activated spleen cells (SC) from infected mice (Lopez et al., 1993), explained this unresponsiveness. By contrast, activated SC from infected mice produced elevated levels of Interferon-γ (IFN-γ) and tumor necrosis factor (TNF) (Tarleton, 1988b; Nabors and Tarleton, 1991). Also, cross-reactive autoantibodies against AGC10 membrane glycoprotein were present in chagasic sera could also suppress the proliferation of human T lymphocytes (Hernández-Munaín et al., 1992) by a mechanism preceding translation of IL-2 and its high-affinity receptor subunits (Kierszenbaum et al., 2002). Moreover, AGC10 was able to inhibit T cell activation, IL-2R, and IL-2 transcription through L-selectin (Kierszenbaum et al., 1999; Alcaide and Fresno, 2004). Other soluble substances, including suppressive cytokines as transforming growth factor-β (TGF-β), IL-4, IL-10, and prostaglandins, released upon contact with parasite-antigens, have also been proposed as the cause of *T. cruzi* immunosuppression (Kierszenbaum, 1982; Tarleton, 1988a; Fernandez-Gomez et al., 1998; Hansen et al., 1998; Pinge-Filho et al., 1999). In particular, IL-12 produced by macrophages in response to infection mediated resistance to *T. cruzi* (Aliberti et al., 1996).

### MDSCs and Infection

It was proposed that inhibition of T cell proliferation in *T. cruzi*-infected mice takes place through IFN-γ and NO secretion (Abrahamsohn and Coffman, 1995). *In vitro* studies identified Th1 cell-derived TNF and IFN-γ as the most important cytokines involved in the killing of intracellular *T. cruzi* through a NO-mediated L-arginine- dependent killing mechanism (Gazzinelli et al., 1992; Muñoz-Fernández et al., 1992b). This was corroborated *in vivo* since anti-IFN-γ administration resulted in a drastic increase in parasitemia and mortality (Torrico et al., 1991; Silva et al., 1992). Other studies showed that NO played a role in host resistance to *T. cruzi* infection in mice (Petray et al., 1993).

We were the first to clearly describe CD11b^+^Gr-1^+^ MSCs (later re-named as MDSCs) in the spleen on infected *T. cruzi* mice that mediated T cell suppression through the production of NO (Goñi et al., 2002). Moreover, this finding of MDSC suppressor activity mediated by NO was the earliest described in the context of any infection. Later we found that M-MDSCs infiltrate target organs like the heart during *T. cruzi* infection (Cuervo et al., 2011).

Since then MDSCs were found in many infections caused by viral, bacterial, and protozoan parasitic pathogens. Thus, among them, human immunodeficiency virus, Cytomegalovirus, hepatitis B, and hepatitis C viruses impaired lymphocyte function. Similar results were found in bacterial infections by *Staphylococcus aureus*, *Francisella tularensis, Mycobacterium* spp., *Klebsiella pneumoniae*, *Helicobacter pylori*, *Escherichia coli*, and polymicrobial sepsis (reviewed in Dorhoi and Du Plessis, 2017).

Medullary or extramedullary myelopoiesis may explain the expansion of MDSCs in other protozoan parasitic infections (reviewed by Van Ginderachter et al., 2010). For instance, in *Leishmania major* infection undifferentiated macrophage-granulocytes mediated susceptibility of BALB/c mice (Mirkovich et al., 1986), but others found that MDSCs were protective (Pereira et al., 2011). However, the genetic background defines MDSC differentiation where *L. major* parasites can modulate the suppressive effect of MDSCs in a strain-dependent manner (Schmid et al., 2014). Besides, in *L. donovani* infection there was enhanced myelopoiesis involving GM-CSF and TNF-α (Cotterell et al., 2000). Other authors also found that *L. donovani* induces hematopoietic stem cells (HSC) expansion and skews their differentiation towards non-classical myeloid progenitors with a regulatory phenotype (Bandyopadhyay et al., 2015), suggesting that emergency hematopoiesis contributes to the pathogenesis of visceral leishmaniasis, as decreased hematopoietic stem cells (HSCs) expansion results in a lower parasite burden (Abidin et al., 2017). Thus, MDSCs in leishmaniosis may play an important role, although it depends on the magnitude of the expansion in different experimental models.

In *Plasmodium chabaudi* nonlethal infection, splenic expansion of CD11b^+^ Gr-1^+^ cells was also observed (Sponaas et al., 2009), and also the emergence of atypical progenitors that had both lymphoid and myeloid potential with a strong bias toward the generation of myeloid cells *in vivo* (Belyaev et al., 2010). Finally, after *Toxoplasma gondii* infection Gr-1+MDSCs played a regulatory protective role (Voisin et al., 2004; Dunay et al., 2008).

### MDSCs in *Trypanosoma cruzi* Infection

**Figure 2:**
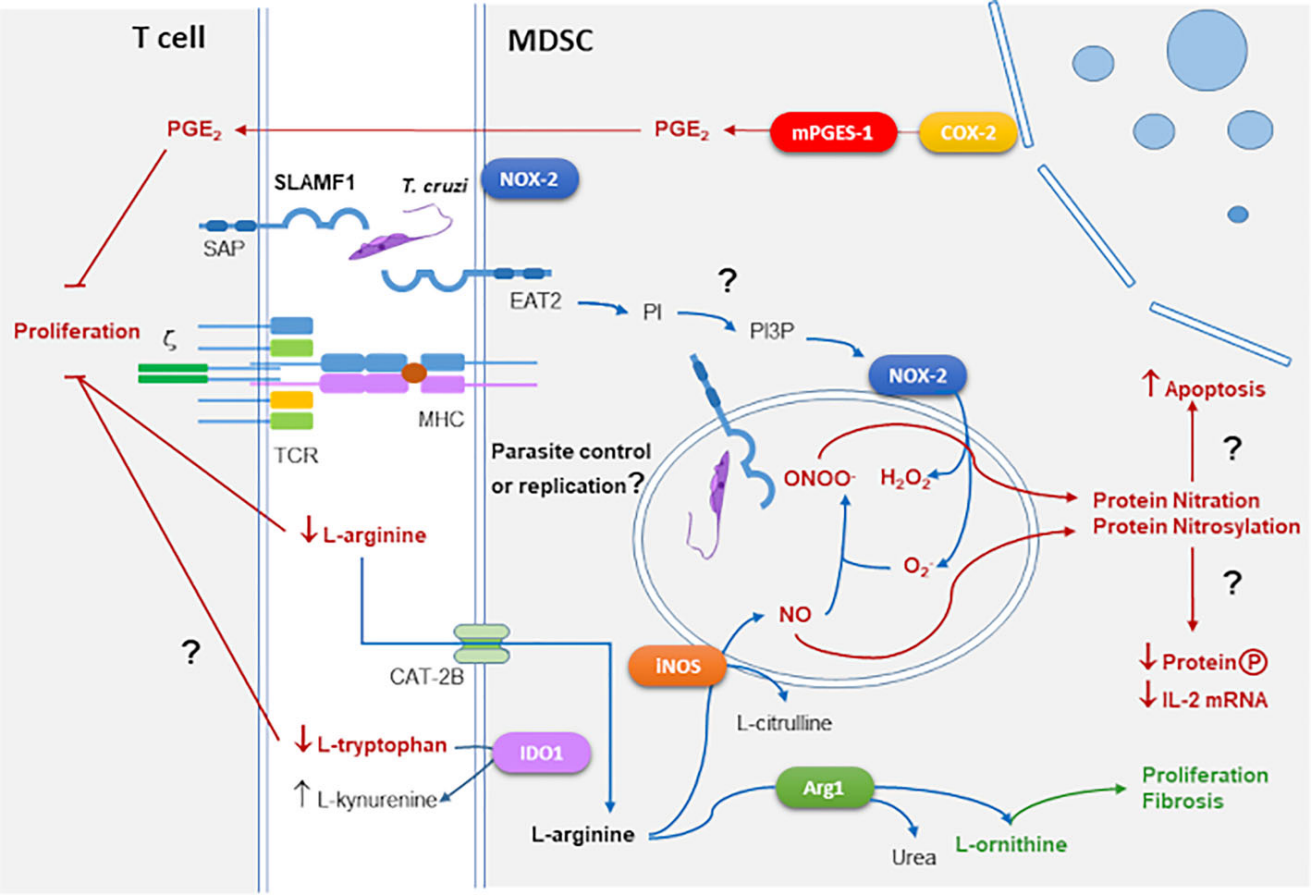
MDSCs suppressive mechanisms in the context of *T. cruzi* infection and SLAMF1 receptor. As outlined in **Figure 1**, enzymes as iNOS, NOX-2, COX-2, and Arg1 mediate suppression in MDSCs. These enzymes are involved in the response against *T. cruzi* infection. In addition, we incorporated the SLAMF1 receptor interacting with *T. cruzi* which through the EAT2 adaptor can trigger the conversion of PI to PI3P activating NOX-2. Question marks indicate those processes that need to be further explored.

Some of the main mechanisms of suppression of T cell proliferation by MDSC in *T. cruzi* infection are outlined in **Figure 2**.

We have described that during acute *T. cruzi* infection of BALB/c mice infected with the Y strain, the cardiac inflammatory infiltrate involves CD11b^+^Ly6C^+^Ly6G^lo^ M-MDSC expressing Arg1 and iNOS, which suppressed T cell proliferation (Cuervo et al., 2008; Cuervo et al., 2011; Carbajosa et al., 2018). Using two mouse strains with different genetic backgrounds and responses to infection, C57BL/6, and BALB/c, M-MDSCs were expanded with greater magnitude in BALB/c mice, which died within one month post-infection. But C57BL/6 mice showed a high percentage of survival, indirectly confirming the relationship with M-MDSC appearance with pathogenicity in acute *T. cruzi* infection. On the other hand, Arocena et al. (2014) found a new facet of MDSCs as regulators of inflammation while reducing parasite burden. They documented a higher number of MDSCs in the spleens and livers of infected BALB/c mice compared with C57BL/6 mice during acute infection. Also, mechanistic studies demonstrated that ROS and NO were involved in the suppressive activity of MDSCs, with high expression of NOX-2 and phosphorylation of signal transducer and activator of transcription-3 (STAT3). Moreover, *in vivo* depletion of MDSCs with 5-fluorouracil (5FU) led to an increased production of IL-6, IFN-γ, and a Th17 response associated with very high parasitemia and mortality in C57BL/6 (Arocena et al., 2014). Thus, the results suggest that the role of MDSCs depends on host and parasite genetic backgrounds, which condition the targeting of different organs during infection. Those authors found that IL-6 signaling leads to the phosphorylation of the STAT3, which in other contexts plays a critical role in the accumulation of immature myeloid cells (Nefedova et al., 2004).

In our studies also the expansion of MDSCs was preceded by higher levels of IL-6 in susceptible mice compared to non-susceptible (Sanoja et al., 2013). In addition, we demonstrated the presence of cardiac M-MDSCs cells in infected BALB/c mice, which significantly contributed to heart leukocyte infiltration, that besides iNOs and Arg1, expressed COX-2 and mPGES-1 and produced PGE_2_, chemokines, and inflammatory cytokines, suggesting that COX-2 is detrimental for the host (Guerrero et al., 2015).

Likewise, we found in infected heart tissue non-suppressor PMN-MDSCs that did not express iNOS nor Arg1 but expressed S100A8/A9 MDSC markers (Cuervo et al., 2011), and thus, they should be classified as MDSC-LC according to the latest nomenclature recommendations (Bronte et al., 2016). S100A8/A9 are involved in leukocyte recruitment and inducing cytokine secretion (Wang et al., 2018), and could be attracting M-MDSCs to heart tissue (Cuervo et al., 2011). Notably, M-MDSCs expansion was linked to local and systemic extracellular L-arg depletion, likely involved in immunosuppression, which reduced NO production by iNOS due to substrate depletion. More importantly, L-arg supplementation to infected mice almost restored plasma L-arg and NO levels, significantly decreased parasitemia, heart parasite burden, and improved clinical status, while increasing mice survival and cardiac performance (Carbajosa et al., 2018). Similar results were recently obtained by others (Bianchini Narde et al., 2021). However, paradoxically, they also found promoted collagenogenesis in the heart muscle tissue suggesting that further analysis of cardiac function is needed to clarify the effect of arginine treatment under the evolution of Chagas cardiomyopathy. On the other hand, L-arg supplementation was beneficial in preventing vertical transmission in infected rats (da Costa et al., 2014).

Interestingly, we found elevated levels of asymmetric+symmetric dimethylarginine (ADMA+SDMA) in heart tissue and plasma of *T. cruzi* infected mice (Carbajosa et al., 2018). ADMA is a product of nuclear proteolysis by arginine methyltransferases (Teerlink, 2005), and is an endogenous inhibitor of iNOS that in combination with L-arg can regulate iNOS activity (Blackwell, 2010). Thus, despite iNOS expression is elevated, its activity could be inhibited by the combination of low L-arg as substrate and high levels of ADMA inhibitor (Carbajosa et al., 2018). Interestingly, a low L-arg/ADMA ratio is a predictor of NO bioavailability and mortality in Dilated Cardiomyopathy, a pathology that resembles Chronic Chagasic Cardiomyopathy (CCC) suggesting a possible relation between L-arg/ADMA levels and CCC (Carbajosa et al., 2018).

In this direction, since Arg1 is induced in M2 macrophages by IL-4 and IL-13 through IL-4Rα, transgenic mice overexpressing IL-13 were much more susceptible to *T. cruzi* infection and this correlated with the increased expression of Arg1, but also iNOS (Abad Dar and Hölscher, 2018). More importantly, the death of IL-13 transgenic animals due to infection was blocked by the administration of Arg1 inhibitors. Thus the authors discuss that IL-4Rα-induced Arg-1 mediates susceptibility to acute experimental Chagas disease by several - mutually not exclusive - mechanisms downstream of Arg-1 including L-arginine depletion in MDSCs and M2 macrophages and polyamine synthesis.

On the other hand, the enzyme IDO, which metabolizes L-tryptophan to L-kynurenine, is critical for host resistance against *T.cruzi* (Knubel et al., 2010). Moreover, the tryptophan metabolite 3−hydroxykynurenine was active against *T. cruzi* and prevented the inflammatory pathology from preventing the clinical symptoms of chronic Chagas disease (Knubel et al., 2011; Knubel et al., 2017). We found by a global metabolomic study that in the experimental *T. cruzi* acute infection circulating and heart tissue levels of L-kynurenine were incremented, but L-tryptophan remained stable (Gironès et al., 2014). Since in our model of acute infection MDSCs there is a huge expansion of MDSCs, tryptophan metabolism in MDSCs infiltrating the heart may be also involved in the immunosuppression.

In addition, inflammatory monocyte-derived dendritic cells (moDCs) were found in the skin of C57BL/6 mice after intradermal inoculation with *T. cruzi* RA strain, produced TNF-α and NO, but also IL-10 and displayed a poor capacity to induce lymphoproliferation. But, ablation of Mo-DCs by treatment with 5FU confirmed their dual role during infection, limiting the parasite load by iNOS-related mechanisms and negatively affecting the development of anti-parasite T-cell response. The authors demonstrated that these cells not only control parasite spreading but also its persistence in the host (Poncini and González-Cappa, 2017). Thus, in the skin mo-DCs may play a regulatory role.

Notably, immunization of BALB/c mice with *T. cruzi* trans-sialidase protected them from infection with the Tulahuen strain of *T. cruzi* and significantly reduced the expansion of PMN-MDSCs (Prochetto et al., 2017). On the other hand, targeting MDSCs by 5FU administration before each dose of trans-sialidase-based vaccine (TSf-ISPA) was able to significantly ameliorate survival and decrease parasitemia levels of TSf-ISPA-vaccinated and infected mice (Gamba et al., 2021). However, the detrimental role of MDSCs in *T. cruzi* infection may be assumed in some models, in others, their depletion results in high mortality, and some questions are raised about whether they should be considered in vaccine design (Cabrera and Marcipar, 2019).

### Host-Derived RNS and ROS in *Trypanosoma cruzi* Infection

Apart from the previously mentioned reports on the role of NO in immune suppression during *T. cruzi* infection, several groups investigated the dual role of NO in controlling parasite burden and as the cause of tissue damage or toxicity. Studies *in vitro* showed the mediation of TNF and INF-γ in the resistance to *T. cruzi* infection in both human (Muñoz-Fernández et al., 1992a) and mouse macrophages (Silva et al., 1995) by increased NO production. Another report *in vivo* infections showed that the production of NO was host-dependent (de Oliveira et al., 1997). Macrophages from mice deficient in the expression of IFN-γ receptor and iNOS showed impaired trypanocidal activity of macrophages (Hölscher et al., 1998). But others reported adverse effects of NO generated by cardiac iNOS during infection that may participate in the pathogenesis of murine chagasic heart disease (Huang et al., 1999), gastrointestinal damage (Ny et al., 1999; Arantes et al., 2004; Ny et al., 2008), heart and spleen damage (Ribeiro et al., 2007), and contribute to neuronal death (Bombeiro et al., 2010). Finally, it was reported that iNOS was not essential to control the infection in mice (Cummings and Tarleton, 2004). Thus, still there are some doubts about the role of NO. This is likely due to the different immunopathogenic mechanisms triggered by different parasite strains in mice as well as the host genetic backgrounds.

There are also divergent results on the role of NOX-2 and ROS during *T. cruzi* infection. Pioneer works showed that ROS was detected in the portion of the plasma membrane of the macrophages to which the parasites attached, and in the membrane which surrounds endocytic vacuoles containing ingested *T. cruzi* parasites (de Carvalho and de Souza, 1987). Also, invasion by *T. cruzi* and an inflammatory milieu affected mitochondrial integrity and contributed to electron transport chain inefficiency and ROS production in cardiomyocytes (Gupta et al., 2009). Moreover, cruzipain, a major *T. cruzi* cysteine protease favored oxidative burst in murine cells (Guiñazú et al., 2010). Bystander effects of heart-infiltrating phagocytes and CD8^+^ T cells resulting in cardiac remodeling in chagasic mice by the action of NOX and ROS were also described (Dhiman and Garg, 2011). More recently, using macrophages deficient in the expression of NLR family pyrin domain containing 3 (NLRP3) it was shown that NLRP3-mediated IL-1β/NFκB activation was dispensable and compensated by ROS-mediated control of *T. cruzi* replication and survival in macrophages (Dey et al., 2014). Moreover, NOX-2 deficiency, compromised CD8^+^ T cell response, leading to increased parasite burden, tissue pathogenesis, and mortality in chagasic mice (Dhiman and Garg, 2014). However, other reports show that *T. cruzi* needs a signal provided by ROS to infect macrophages (Goes et al., 2016). In the same direction, cyclophilin 19 released in the host cell cytosol by *T. cruzi* mediates the increase of ROS, required to boost parasite proliferation in mammalian hosts (Dos Santos et al., 2021).

On the other hand, ONOO^-^, resulting from the combination of NO and ${\text{O}}_{2}^{-}$, acts as an intraphagosomal cytotoxin against pathogens, while microbial peroxiredoxins facilitate infectivity *via* decomposition of macrophage-derived ONOO^-^ (Alvarez et al., 2011). Moreover, it was shown that ${\text{O}}_{2}^{-}$ did not play a critical role in the control of the parasite, but it was important to prevent blood pressure decline during infection with *T. cruzi* (Santiago et al., 2012).

## Role of SLAMF1 Receptor on ROS Generation and MDSC Expansion During *Trypanosoma cruzi* Infection

The SLAMF1 receptor was shown to play an important role in the context of *T. cruzi* infection (**Figure 2**). The SLAM family of receptors is composed of 9 members with a similar structure. SLAMF1-7 genes are located in the same cluster in chromosome 1 and contain intracellular immuno-receptor tyrosine-based activation motif (ITAM). But SLAMF8-9, located nearby both in human and mouse, lack intracellular ITAM motifs. SLAM-family receptors expressed by different immune cells can form homophilic interactions between them, recruiting adaptor proteins as SLAM-associated adaptor (SAP) in T cells that enhance IL-4 and IL-13 production (Davidson et al., 2004). SLAMF1 (CD150), expressed also in HSCs, is a self-ligand that mediates IL-4 production after interaction of an antigen-presenting cell (APC) with a CD4^+^ T cell. It also regulates natural killer cell (NKT) differentiation after interaction between double-positive T cells (Sintes and Engel, 2011). In the context of bacterial infection, the interaction of OmpC/F+*E. coli* with SLAMF1 is required for macrophage phagocytosis and phagosome localization where EWS/FLI1 activated transcript 2 (EAT2) enhances Phosphatidylinositol-3-phosphate (PI3P) production, activation of NOX-2 and ${\text{O}}_{2}^{-}$ production, which besides antimicrobial activity, is a signaling molecule that modulates cell motility and phagocytosis, thus, in the absence of SLAMF1 the phagocytic process of Gram- bacteria is compromised (van Driel et al., 2016). However, SLAMF1 was not required in infections with a Gram+ bacteria like *Staphylococcus aureus* (Berger et al., 2010).

We found that BALB/c mice deficient in the expression of SLAMF1 (*Slamf1^-/-^*) survived the infection with a lethal dose of the *T. cruzi* Y strain for which, as observed before, susceptible wild type (WT) BALB/c succumbed to the infection (Calderón et al., 2012). In addition, *Slamf1^-/-^* macrophages became more resistant to the infection with the same strain. More recently, we described increased resistance of *Slamf1^-/-^* macrophages to the infection with other parasite strains (Dm28c, M6421, 10R26, and Bug2148) except for one (VFRA) (Poveda et al., 2020). This correlated with the increased expression of NOX-2 expression and ROS production in *Slamf1^-/-^* macrophages infected with all the strains except VFRA in comparison to the WT. These opposing strain-dependent roles of SLAMF1 in infectivity and ROS production may explain some of the mentioned discrepancies in the chapter above about the role of ROS in *T. cruzi* infection. Moreover, *Slamf1^-/-^* mice infected with the Y strain showed a shift from MDSC expansion to M1 polarization compared to the WT (Poveda et al., 2020), suggesting that SLAMF1 may have a role in emergency myelopoiesis and possibly mediating MDSCs expansion, a possibility that should be explored in the future.

## Conclusions

In *T. cruzi* infection, immune suppression was described in seminal studies decades ago. First suppression was thought to depend on particular molecules until NO was identified as one of the suppressor mediators in MDSCs. The mechanism of suppression mediated by NO and other RNS is the toxicity caused by protein nitrosylation and/or nitration, which also can cause tissue damage and vasodilation. On the other hand, is essential as an antimicrobial defense in phagolysosomes. Thus, NO has many roles. In *T. cruzi* infection, the last has been questioned since it was not necessary for controlling infection in certain models. This might be due to particular combinations of parasite and mouse strains, or the development of compensatory mechanisms that help to control infection in the absence of NO production.

In our hands, NO protects from *T. cruzi* infection, but the expansion of MDSCs that express iNOS is detrimental for infected mice with immunosuppressive effects. In addition, MDSCs expansion is associated with L-arg depletion by Arg1, which causes further suppression. It will be interesting to study the effect of MDSCs expansion inhibition in this model of experimental infection.

Another antimicrobial mechanism in MDSCs is ROS production by NOX-2. Again the role of NOX-2 in *T. cruzi* infection is controversial. Thus, some reports, like ours, link NOX-2 and resistance to infection, but others claim that ROS produced by NOX-2 is needed for parasite proliferation. We believe that an explanation could be that parasites with strong antioxidant defenses can proliferate in the presence of low levels of ROS, but likely they succumb when ROS levels are high.

In humans, the expression of Arg1 by myeloid cells is thought to be restricted to granulocytes. However, there is some evidence that human monocytes/macrophages can express Arg1. Thus, it will be interesting to perform studies to evaluate the plasma levels of L-arg in humans with *T. cruzi* acute infection and/or in vertical transmission by infected pregnant mothers. If L-arg levels turn out to be low in acute/chronic Chagas disease patients, they could receive drug therapy in combination with L-arg supplementation to eliminate the parasite more efficiently. This may enable in the future to reduce the dose of conventional antiparasitic drugs, which present many secondary effects due to toxicity. Although the excessive expansion of MDSCs is host detrimental, their complete depletion could have adverse effects according to the reported results. In this direction, blocking SLAMF1 interaction with the parasite might be beneficial for the host by skewing MDSCs expansion. Thus, the identification of SLAMF1 parasite ligands can lead to the development of new therapies.

## Author Contributions

MF and NG wrote the manuscript. All authors contributed to the article and approved the submitted version.

## Funding

This work was supported by “Ministerio de Economía y competitividad/FEDER” (MINECO/FEDER) SAF2015-63868-R and “FEDER/Ministerio de Ciencia, Innovación y Universidades Agencia Estatal de Investigación” (MICINN/FEDER) PGC2018-096132-B-I00 to NG, SAF2016-75988-R (MINECO/FEDER) to MF); “Red de Investigación de Centros de Enfermedades Tropicales” (RICET RD12/0018/0004 to MF); Comunidad de Madrid (S-2010/BMD-2332) to MF; and Institutional grants from “Fundación Ramón Areces” and “Banco de Santander”.

## Conflict of Interest

The authors declare that the research was conducted in the absence of any commercial or financial relationships that could be construed as a potential conflict of interest.

## Publisher’s Note

All claims expressed in this article are solely those of the authors and do not necessarily represent those of their affiliated organizations, or those of the publisher, the editors and the reviewers. Any product that may be evaluated in this article, or claim that may be made by its manufacturer, is not guaranteed or endorsed by the publisher.
